# Resveratrol mitigates the oxidative stress mediated by hypoxic-ischemic brain injury in neonatal rats via Nrf2/HO-1 pathway

**DOI:** 10.1080/13880209.2018.1502326

**Published:** 2018-11-21

**Authors:** Yan Gao, Rongrong Fu, Jue Wang, Xue Yang, Lulu Wen, Juan Feng

**Affiliations:** Department of Neurology, Shengjing Hospital Affiliated Hospital of China Medical University, Shenyang, Liaoning, China

**Keywords:** Lipid peroxidation, inflammatory markers, infarct area, oedema, neurotherapeutic

## Abstract

**Context:** Hypoxic-ischemic encephalopathy (HIE) has a high morbidity and mortality rate. Resveratrol possesses numerous biological properties including antioxidant, anti-inflammatory and neuroprotective activities.

**Objective:** The current experiment investigates the neuroprotective efficacy of resveratrol (RESV) against HIE by modulating Nrf2/HO-1 pathway in neonatal rats.

**Materials and methods:** Seven-day-old pups (*n* = 48) were divided into four groups. Group-I rats receiving 2% DMSO saline (sham), group-II rats underwent unilateral carotid artery ligation and hypoxia (92% N_2_ and 8% O_2_) for 2.5 h (hypoxia-ischemia; HI), group-III and IV rats received 20 (RESV 20 + HI) or 40 mg/kg (RESV 40 + HI; group-IV) of RESV via intraperitoneal injection (ip), respectively, for 7 days prior to HI induction.

**Results:** Pre-treatment with RESV (20 or 40) markedly reduced (*p* < 0.01) the cerebral oedema (86.23–71.26 or 65.24%), infarct area (33.85–19.81 or 14.30%), lipid peroxidation products, inflammatory markers [IL-1β 186–110 or 82; IL-6 255–146 or 103; TNF-α 310–204 or 137; NF-κB 205–115 or 91) p65 subunit] and significantly restored (*p* < 0.01) the antioxidative status by enhancing the activities of glutathione peroxidase (GPx) 5.22–6.49 or 7.78; catalase (CAT) 51–55 or 59, superoxide dismutase (SOD) 2.5–3.05 or 3.25; through marked upregulation (*p* < 0.01) of heme oxygenase 1 (HO-1) 0.65–0.69 or 0.73; and nuclear factor erythroid 2 related factor 2 (Nrf2) 0.73–0.86 or 0.91.

**Discussion and Conclusions:** RESV displays its neurotherapeutic potential via upregulating the protein expression of Nrf2 and HO-1 signalling pathway and thereby attenuates oxidative stress and inflammatory response in HI-induced neonatal rats.

## Introduction

Hypoxic-ischemic brain injury (HIBI) is the principal contributor to several types of neurological impairment (Long and Brandon 2007). Approximately 40–60% of HIBI affected premature infants succumb to HIBI by the age of 2. Every two newborns per 1000 full term births are affected by neonatal hypoxic-ischemic encephalopathy (HIE) (Kurinczuk et al. 2010; Zhang et al. 2016). The crucial pathophysiological event in HIE is the restriction of cerebral blood flow, which reduces oxygen supply to the brain cells and eventually leads to a collapsed electron transport chain (energy production) by a multifaceted cascade of biochemical events such as enhanced oxidant stress, inflammation, excitotoxicity and delayed cell death (Allen and Brandon 2011; Lu et al. 2016). HIE as a clinical condition has two fundamental pathophysiological events: HI and reperfusion (Ten and Starkov 2012). The present treatment regimen or procedures for HIE includes stem cell transplantation, anticonvulsants (anti-epileptic) drugs, and hypothermic treatments. However, the above mentioned treatment regimen or procedures are expensive and associated with few adverse effects (West et al. 2007; Zanelli et al. 2009).

The antioxidants play a pivotal role in maintaining the balance between free radical generation (oxidants) and antioxidant activities and thus abolish the condition called oxidative stress (imbalance between free radicals and antioxidants) (Gitto et al. 2013). The neonatal brain is highly vulnerable to oxidative stress (damage), more than the adult, owing to lower levels of antioxidants, high oxygen consumption rate, high contents of unsaturated fatty acids, high water content, low myelinization and availability of redox-active iron (Buonocore and Groenendaal 2007). Certain studies have shown a direct association between the degree of hypoxia and the severity of oxidative stress owing to free radical production during hypoxia in the neonatal period (Perrone et al. 2010; Lu et al. 2012). Hence, a logical strategy for treating HIE in neonatal rats is to augment the antioxidative status by antioxidant agents. Previous studies have proven the positive impact of antioxidant agents against HIE (West et al. 2007; Ping et al. 2010).

Resveratrol (RESV; tri-hydroxy stilbene) is a naturally occurring non-flavonoid polyphenolic compound belonging to the phytoalexin superfamily. It has two aromatic rings with three free hydroxyl groups which contributes to its free radical scavenging and antioxidant activities (Yousuf et al. 2009). The primary dietary sources of RESV are red wine/red grapes, soybeans, and pomegranates (Liu et al. 2007). RESV has been used to treat various ailments including diabetes, cardiovascular diseases, cancers and neurological diseases (Karalis et al. 2011; Feng et al. 2016; Sadi and Konat 2016). Resveratrol may exert its neuroprotective activity by its antioxidant, anti-inflammatory, anti-apoptotic properties via activating sirtuin 1 (SIRT1) and thereby positively modulate the mitogen-activated protein kinase (MAPK-p38) and Akt/Phosphoinositide-3-kinase (PI3K) signalling pathways and subsequently downregulating the expression of glycogen synthase kinase-3β and cyclic adenosine monophosphate (cAMP) response element binding protein to protect the neuronal cells (Shin et al. 2012; Meng et al. 2015; Kodali et al. 2015). Furthermore, RESV could readily cross through the blood-brain barrier (BBB), which makes it as a suitable candidate for a neuroprotective drug (Wang et al. 2002).

A transcriptional factor called nuclear factor erythroid 2 related factor 2 (Nrf2) has been reported to abolish oxidative stress by upregulating cytoprotective factors namely NADPH quinone oxidoreductase 1 (NQO1), heme oxygenase 1 (HO-1) as well as antioxidants like glutathione (family) and catalase (CAT) via activating the antioxidant response element (ARE) (Ren et al. 2011; Zhang et al. 2014). Guo et al. (2014) also demonstrated that Nrf2 might play a pivotal role in activating the endogenous defense system and thus it protects the neural cell from an ischemic brain injury. Numerous studies have shown the neuroprotective effects of resveratrol in various animal models via modulating multiple signalling pathways (Shin et al. 2012; Bastianetto et al. 2015). However, the neuroprotective potential of numerous phyto-components is demonstrated by positively modulating Nrf2/HO-1 signalling pathway (Ping et al. 2010; Peng et al. 2013) in models of ischemia. Hence, the current study was framed to examine the possible association of Nrf2/HO-1 signalling pathway with the neuroprotective activity of resveratrol against HIE in neonatal rats.

## Materials and methods

### Drugs and reagents

Resveratrol, protease lytic buffer, and bromophenol blue were purchased from Sigma (MO, USA). Toluidine blue, paraformaldehyde, glycerol, 2,3,5-tritriphenyl-tetrazolium chloride (TTC), sodium hydroxide, potassium chloride, hydrogen peroxide, isoflurane, phosphate buffered solution, xylene and 3,3′-diaminobenzidine (DAB) were bought from Kangchen Biotechnology (Shanghai, China).

### Neonatal rats

A total of 48 seven-day-old (P7) Sprague–Dawley rat pups (*n* = 48; weighing 17–21 g) were procured from the local Animal merchant (Liaoning, China). Neonatal rats were kept at room temperature (22 °C–25 °C; 65% humidity) in a steel cage with full access to water and standard food. The ethical committee board of Shengjing Hospital, Affiliated to China Medical University (Liaoning, China; reg. no. 11/2014/SH-CMU153) approved the animal experiment of the present study, which conformed to the guidelines from National Institute of Health (NIH; MD, USA) for the care and use of experimental animals.

### Induction of brain injury by HI

Rat pups [14-day old; after 7 days of pretreatment with RESV in RESV 20 + HI and RESV 40 + HI group (group III and IV) or saline in sham and HI group (group I and II)] were anesthetized with 2% isoflurane and checked for the paw pinch reflex to confirm a sufficient level of anaesthesia before surgery. All pups were subjected to unilateral HI as reported previously (Li et al. 2012) with slight modifications. In brief, anesthetized pups underwent unilateral common carotid ligation by silk surgical suture. After a recovery period of 1 h, pups were moved to a hypoxia chamber (8% O_2_ and 92% N_2_), which was maintained at 37 °C (assistance of water bath) for 2.5 h. Then, all pups were returned to their respective cage (parental fed cages) and subsequently injected (intraperitoneally; ip) with saline containing 2% dimethyl sulfoxide (DMSO) in the sham control group or resveratrol in treatment groups (RESV 20 + HI and RESV 40 + HI group). In the sham rats, the carotid artery was not ligated, and animals were exposed to normal atmospheric conditions.

### Experimental protocol

For the current study, a total of 48 rats were used, but four rats died during HI insult. The mean mortality rate during HI induction was 10–20%, as indicated previously by Li et al. (2012). The rat pups were arbitrarily segregated into four groups: In groups I and II, rats received saline containing 2% DMSO ip for 7 days [sham control group (*n* = 12)] and rats subjected to HI insult group [HI group (*n* = 10)], and in groups III and IV, pups received 20 or 40 mg/kg RESV dissolved with 2% DMSO ip (RESV 20 + HI and RESV 40 + HI groups; *n* = 11), respectively, for seven consecutive days before HI induction. When the rats were 15 days old, they were sacrificed (pentobarbital sodium 50 mg/kg) and brain was immediately removed and fixed in 4% paraformaldehyde for morphological analysis as well as for immunohistochemistry (six rats from each group). The cerebral cortex homogenate (pooled) was prepared using Sigma lysis phosphate buffer (Sigma-Aldrich; MO, USA) and used for biochemical and molecular analysis (remaining rats from each group).

## Morphological studies

### Examination of infarct area (size)

The cerebral infarct area were evaluated using 2,3,5-tritriphenyl-tetrazolium chloride (TTC) staining procedure as described previously (Li et al. 2012). In brief, after HI induction, pups were sacrificed, and brain was immediately removed and frozen before sectioning. Then, four coronal sections (2 mm size) were obtained using ultra-microtome (Leica Microsystems, Wetzlar, Germany). Followed by staining the coronal sections with 0.1% TTC at 37 °C for 5 min, and each section was rinsed with PBS. The image of infarct area captured using a CP 5700 (Nikon Corporation, Tokyo, Japan) were analyzed using Image J software (NIH; MD, USA) and the following formula was used to evaluate the infarct size (area):

Infarct area=[Total infarct area/Whole brain section]×100%

### Determination of cerebral oedema

Cerebral oedema was determined by the method of Mdzinarishvili et al. (2007), with slight modification. The dry and wet weight of brain samples were determined to assess the cerebral oedema. A vacuum drying procedure was used for determining dry weight of brain as indicated by Sebastiani et al. (2017).

Cerebral oedema=[Wet weight−dry weight]/wet weight×100%

### Morphological changes monitored by nissl staining

The 4-µm coronal sections were obtained from frozen brain sample by ultra-microtome-Leica RM2235 (Leica Biosystems; Wetzlar, Germany). The coronal section was initially hydrated with 1% toluidine blue solution at 40 °C for 10 min followed by the addition of double distilled and dehydrated with PBS and blocked with 0.3% Triton X100 and subsequently stained with cresyl violet stain. A picture of each cortex sections was captured using a Nikon CP 5700 (Nikon Corporation; Tokyo, Japan) and analyzed by a computerized imaging system integrated with Image J software (NIH; MD, USA) to examine Nissl bodies.

## Biochemical analysis

### Lipid peroxidation and antioxidant status

The levels of superoxide dismutase (SOD; A001 SOD detection kit), catalase (CAT; A007 CAT detection kit), glutathione peroxidase (GPx; A017 glutathione detection kit) and lipid peroxidation product malondialdehyde (MDA; A003 MDA detection kit) levels were assayed using a commercial kit from Nanjing Jiancheng Bioengineering Institute (Nanjing, China) based on the suppliers protocol. The Pierce protein assay kit was used to determine the protein concentration (Thermo Fisher Scientific Inc., MA, USA).

### Assessment of inflammatory markers

Concentration of various proinflammatory cytokines like Interleukin one beta (IL-1β), Interleukin six (IL-6), tumour necrosis factor alpha (TNF-α), nuclear factor kappa B (NF-κB) p65 subunit in cerebral homogenate were assessed by commercially available ELISA kits in accordance to the suppliers instructions (Thermo Fisher Scientific Inc., MA, USA). Likewise, the levels of NF-κB p65 subunit in cerebral cortex homogenate (nuclear fraction using Nuclear/Cytosolic fraction kit from Biovision Inc., CA, USA) was measured using the ActivELISA kit from Imgenex (CA, USA).

### Immunoblot analysis

For the analysis of Nrf2 (nuclear fraction) and HO-1 (cytosolic fraction) of the cerebral cortex, the Nuclear/Cytosolic fraction kit (Biovision Inc., CA, USA) was used based on suppliers protocol. Protein (40 µg/lane) were separated using 10% SDS-PAGE and then electrotransferred onto a nitrocellulose membrane (Thermo Fisher Scientific, Inc.) using a semi-dry blotting system from GE Healthcare (Chalfont, UK). The nitrocellulose membrane was blocked with Tween 20, Tris-buffered saline solution (TBS) and 5% skimmed milk and incubated with primary antibody at 4 °C for overnight. Antibodies employed for this study are as follows: Polyclonal rabbit anti-Nrf2 antibody (1:500 dilution; G921a Promega Corp., Madison, WI, USA), Polyclonal rabbit anti-HO-1 antibody (1:800 dilution; CD143 Stressgen Biotechnologies, Victoria, BC, Canada) and rabbit polyclonal anti-rat β-actin (1:500 dilution; ZGB24 Zhongshan Goldenbridge Biotechnology, Beijing, China) or polyclonal anti-rabbit-histone H3 (1:1200 dilution; ZGHH3 Zhongshan Goldenbridge Biotechnology). Subsequently, samples were probed with a goat anti-rabbit horseradish peroxidase (HRP) conjugated secondary antibody (1:10,000 dilution; ab6721; Abcam, Cambridge, UK) in TBS at room temperature for 1 h with DAB (visualization). The absorbance was quantified using an enhanced chemiluminescence image analyzer with Image J software (NIH; MD, USA).

### Immunohistochemistry (IHC)

Cerebral cortex sections (5 µm) were prepared as indicated before in morphological analysis section. These cerebral sections were washed with water (hydrated) and incubated with 3% hydrogen peroxide at room temperature for 10 min. Followed by washing with PBS and blocked with BCA solution for 30 min and probed with antibodies: Polyclonal rabbit anti-Nrf2 antibody (1:500 dilution; G921a; Promega Corp., Madison, WI, USA), Polyclonal rabbit anti-HO-1 antibody (1:800 dilution; CD143 Stressgen Biotechnologies, Victoria, BC, Canada) for overnight at 37 °C and finally incubated with a goat anti-rabbit horseradish peroxidase (HRP)-conjugated secondary antibody (1:10,000 dilution; ab6721; Abcam, Cambridge, UK) in TBS at room temperature for 1 h with 0.05% DAB (visualization) and 0.01% hydrogen peroxide. Then, the cerebral sections were counterstained using hematoxylin and mounted onto the microscopic slide. Photomicrographs were captured at a magnification of ×400 using a confocal microscope (LSM710; Zeiss, Oberkochen, Germany), and Image J software (NIH; MD, USA) was used to quantify positively stained immunoreactive cells.

### Statistical analysis

Data are expressed as the mean ± standard deviation (*SD*). The statistical difference between the experimental animals was measured using one-way analyses of variance (ANOVA) and followed by Tukey’s *post hoc* multiple comparisons test. All the statistical analyses were carried out with the help of SPSS software (ver 21 from International Business Machines, Corp., NY, USA). A *p*-value less than 0.05 was deemed as statistically significant.

## Results

### Pre-treatment with RESV reduces the cerebral infarct area in rats after HI

**Figure 1.**
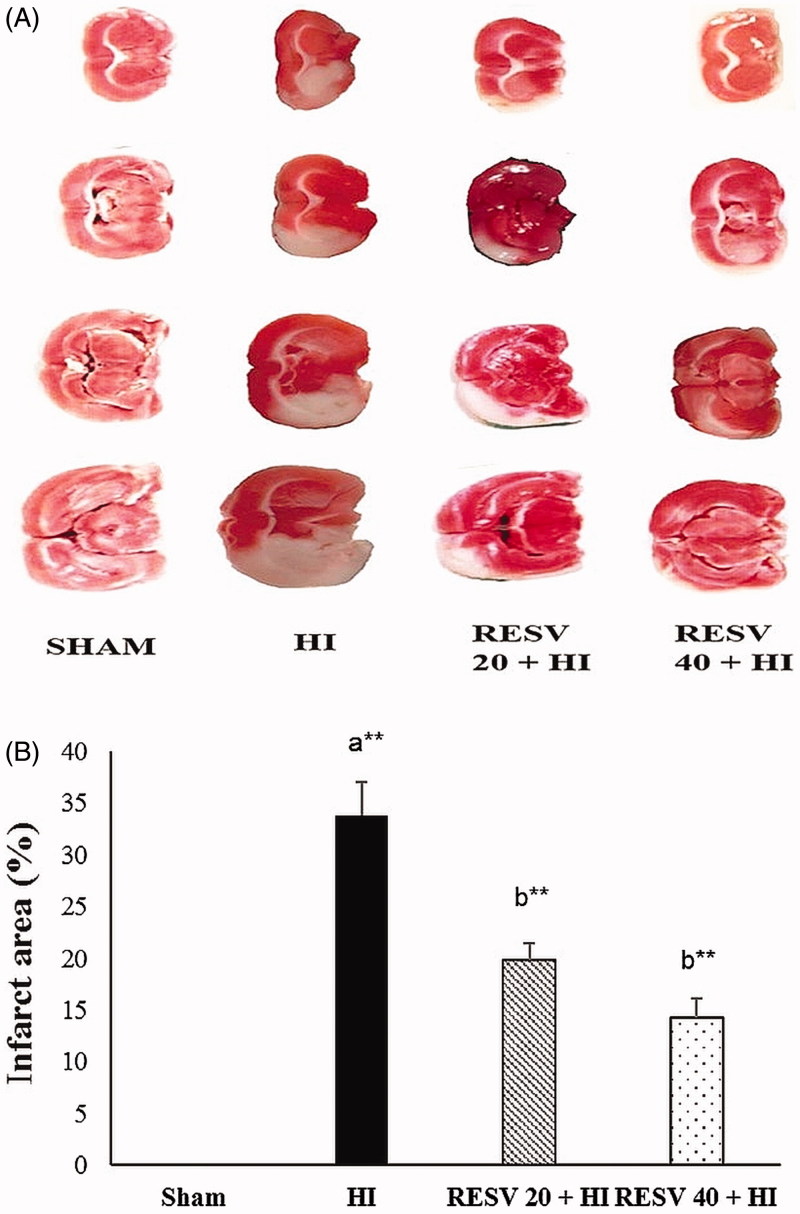
Efficacy of RESV on the cerebral infarct area in experimental neonatal rats. Images of coronal transections stained with TTC illustrate numerous white patches which represent the ischemic area (affected area) (HI group; 1B), whereas the normal tissues were red. Data are expressed as the mean ± standard deviation (*SD*). *p* value: *p < 0.05, **p < 0.01, Where ‘a’ represent the comparison with the sham control group; ‘b’ represent the comparison with the HI-insulted group.

Cerebral infarct area/size in pups brain was assessed using TTC stain (Figure 1). In the HI group, the cerebral infarct area (white patches) was significantly (*p* < 0.01) elevated (33.85 ± 3.15%) more than the sham control group. Whereas, the RESV-treated groups (20 and 40 mg/kg) showed the cerebral infarct area of 19.81 ± 1.65% and 14.30 ± 1.81%, respectively, thus indicating that RESV could considerably suppress the HI-induced increased infarct area (brain damage-white patches).

### RESV pretreatment reduces HI-induced cerebral oedema in rats

**Figure 2.**
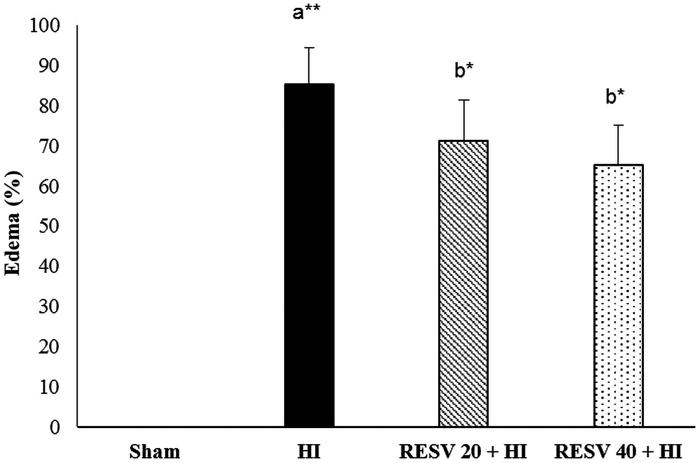
Efficacy of RESV on the cerebral oedema in experimental neonatal rats. Data are expressed as the mean ± standard deviation (*SD*). Data are expressed as the mean ± standard deviation (*SD*). *p* value: *p < 0.05, **p < 0.01, Where ‘a’ represent the comparison with the sham control group; ‘b’ represent the comparison with the HI-insulted group.

The percentage of cerebral oedema in the HI insult group was significantly (*p* < 0.01) escalated (85.23 vs. 0% in the sham group). Pre-treatment with RESV (20 and 40 mg/kg) significantly reduced (*p* < 0.05) the cerebral oedema to 71.26 and 65.24%, respectively, thereby conferring its neuroprotective activity by reducing oedematic condition (water content) as compared with those in the HI group (Figure 2).

### RESV has neuroprotective effects in the brains of rats subjected to HI with increased Nissl bodies

**Figure 3.**
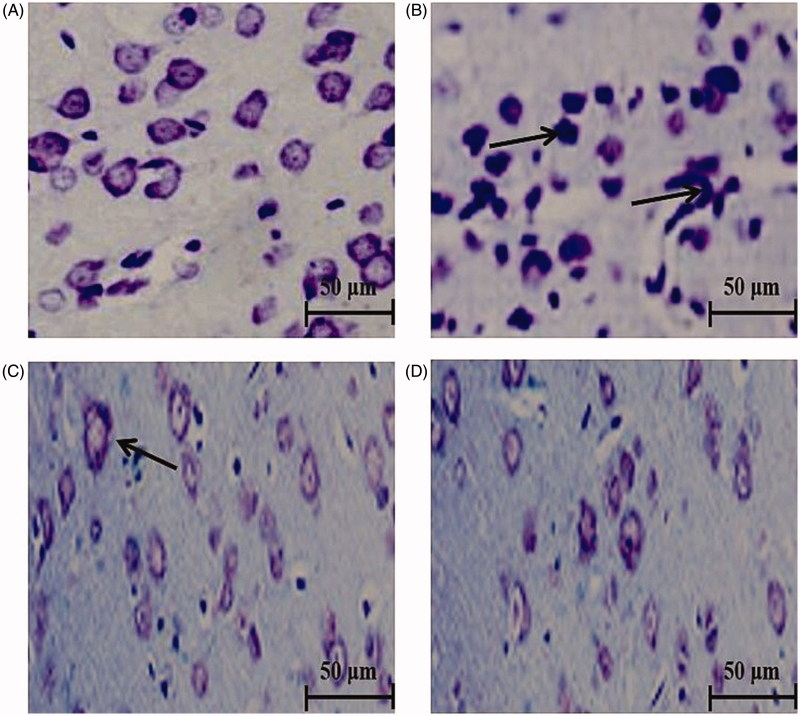
Efficacy of RESV on the cerebral sections stained with Nissl stain (magnification, ×400) in experimental neonatal rats. Cerebral section of sham control rats (A) shows the normal architecture with many Nissl bodies. Several neuro-morphological changes were noted in hypoxia-ischemia-induced rats (B) with increased pyknotic neurons (indicated by arrows) and less Nissl bodies. Pre-treatment with RESV at 20 (C) and 40 mg/kg (D) for 7 days increased a number of Nissl bodies (viable neurons). Scale bar: 50 µm.

Nissl bodies are widely employed to detect viable neurons (Figure 3). Cerebral sections of sham control rats showed the normal typical architecture with numerous prominent Nissl bodies (Figure 3A). Extensive neuronal (morphological) changes were noted in the sections of HI-insulted rats with increased pyknotic neurons, interstitial oedema and less Nissl bodies (Figure 3B). In comparison with the HI group, the RESV pretreatment groups exhibited a markedly better histological structure of neurons with an increased number of Nissl bodies (more viable neurons) and thus indicating the neuroprotective property (Figure 3C and D).

### RESV has antioxidant effects in the brains of rats subjected to HI

Levels of various antioxidant enzymes and lipid peroxidative products (MDA) were determined to assess whether RESV pretreatment would protect the brain of HI-induced rats against oxidative stress (Table 1). In the HI group, the activities of GPx, SOD and CAT were markedly diminished as compared with those in the sham control group, whereas MDA was substantially increased (*p* < 0.01). Pre-treatment with RESV 20 (*p* < 0.05) or 40 (*p* < 0.01) significantly improved the activities of GPx, SOD and CAT as well as remarkably decrease the MDA levels in brain tissues.

**Table 1.** Effect of RESV on the levels of cerebral lipid peroxidation products and antioxidant activity in experimental neonatal rats.

Group	GPx (µg/mg protein)	SOD (U/mg protein)^a^	CAT (U/mg protein)^b^	MDA (nmol/mg protein)
Sham-control	8.53 ± 1.05	3.45 ± 0.28	62.80 ± 5.05	0.48 ± 0.07
HI induced	5.22 ± 0.63a**	2.55 ± 0.18a**	51.24 ± 4.46a**	0.82 ± 0.04a**
RESV 20 + HI	6.49 ± 0.5b*	3.05 ± 0.15b*	55.23 ± 6.34b*	0.69 ± 0.06b*
RESV 40 + HI	7.78 ± 0.75b**	3.25 ± 0.15b**	59.74 ± 6.34b**	0.57 ± 0.07b**

Data are expressed as the mean ± standard deviation (*SD*). *p* value: *p < 0.05, **p < 0.01, Where ‘a’ represent the comparison with the sham control group; ‘b’ represent the comparison with the HI-insulted group.

^a^U (One unit) represents the amount of SOD required for inhibiting 50% of O_2_^−^ at 550 nm.

^b^U (One unit) represents the amount of CAT required for inhibiting 50% of H_2_O_2_ at 405 nm.

RESV: Resveratrol; HI: hypoxia-ischemia; U: units; SOD: superoxide dismutase; CAT: catalase; GSH: glutathione; MDA: malondialdehyde.

### RESV inhibits inflammation in the brains of rat pups subjected to HI

The anti-inflammatory effects of RESV after HI induction were assessed by evaluating the levels of inflammatory markers (pro-inflammatory cytokines) like IL-6, IL-1β and TNF-α as well as NF-κB free p65 subunit (Table 2). A pronounced increase (*p* < 0.01) in the levels of inflammatory markers (IL-6, IL-1β, TNF-α and NF-κB free p65) was observed in HI group than sham control group. Treatment with RESV significantly inhibited (*p* < 0.01) the HI-induced increases of these inflammatory markers. However, the anti-inflammatory effect of the 40 mg/kg of RESV was better than that of 20 mg/kg of RESV.

**Table 2.** Effect of RESV on the concentration of cerebral inflammatory markers in experimental neonatal rats.

Group	TNF-α (ng/mg protein)	IL-β (ng/mg protein)	IL-6 (pg/mg protein)	NF-κB p65 (pg/mg protein)
Sham-control	113.49 ± 12.30	60.35 ± 5.78	80.53 ± 9.11	76.53 ± 6.34
HI induced	310.75 ± 18.10a**	186.52 ± 15.06a**	255.38 ± 22.02a**	205.38 ± 21.02a**
RESV 20 + HI	204.12 ± 21.16b**	110.68 ± 12.10b*	146.45 ± 13.07b*	115.75 ± 11.03b**
RESV 40 + HI	137.28 ± 13.34b**	82.74 ± 9.23b**	103.46 ± 8.09b**	91.36 ± 10.73b**

Data are expressed as the mean ± standard deviation (*SD*). *p* value: *p < 0.05, **p < 0.01, Where ‘a’ represent the comparison with the sham control group; ‘b’ represent the comparison with the HI-insulted group.

RESV: Resveratrol; HI: hypoxia-ischemia; TNF: tumour necrosis factor; IL: interleukin; NF-κB: nuclear factor kappa B.

### RESV enhances protein expression of nuclear Nrf2 and cytosolic HO-1 in the cerebral cortex of rats

**Figure 4.**
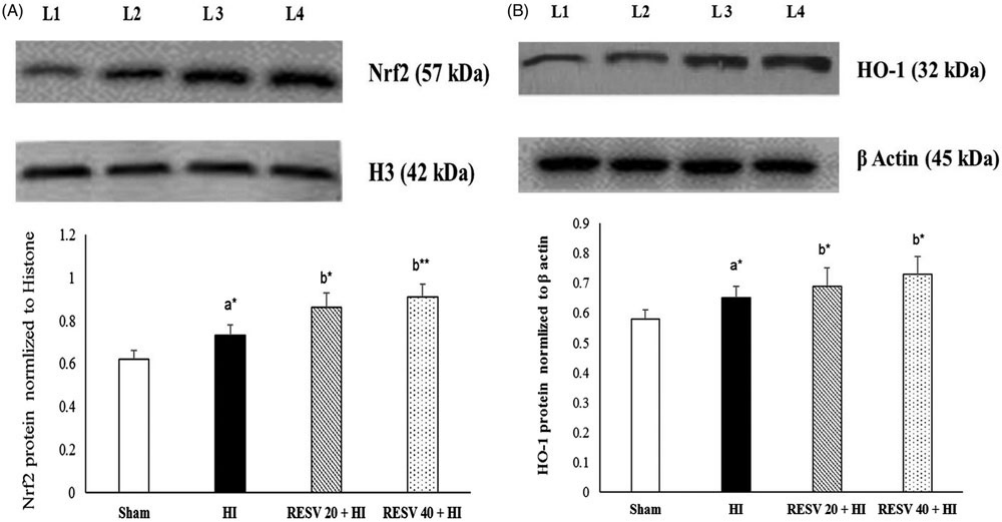
Efficacy of RESV on the protein expression of nuclear Nrf2 (A) and cytosolic HO-1 (B) in the cerebral cortex of experimental neonatal rats. Data are expressed as the mean ± standard deviation (*SD*). *p* value: *p < 0.05, **p < 0.01, Where ‘a’ represent the comparison with the sham control group; ‘b’ represent the comparison with the HI-insulted group. Lanes 1 (L1): sham control group; Lane 2 (L2): HI group; Lane 3 (L3): RESV 20 + HI group; Lane 4 (L4): RESV 40 + HI group.

Immunoblot analyses were preferred to check the effects of RESV on the protein expression of cytosolic HO-1 and nuclear Nrf2 (Figure 4). The protein expression of Nrf2 (nuclear fraction) and HO-1 (cytosolic fraction) in the cerebral cortex were remarkably upregulated (*p* < 0.05) in HI insulted rats on equivalence to the sham group. Of note, pretreatment with RESV (20 or 40) further upregulate the protein expression of cytosolic HO-1 and nuclear Nrf2 after HI insult.

### RESV improve the immune-reactivity of Nrf2 and HO-1 in the cerebral cortex of neonatal rats

**Figure 5.**
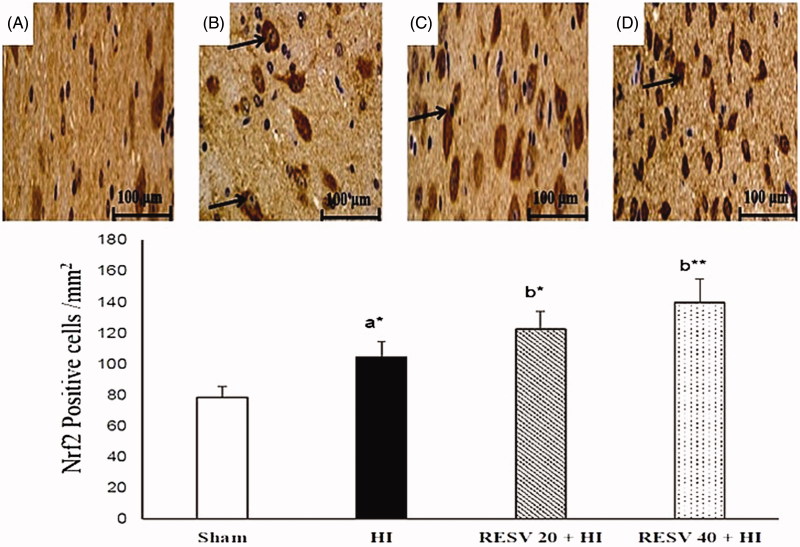
Efficacy of RESV on the immune-reactivity of Nrf2 (A–D) in the cerebral region of experimental neonatal rats. Relatively less positively stained nuclear Nrf2 was observed in the sham control rats (A). Increased positive staining of cells for nuclear Nrf2 was noted in cerebral tissues (infarct regions) of the HI-induced brain (B). Furthermore, increase in the staining for nuclear Nrf2 was observed (indicated by arrows) in both RESV 20 + HI (C) and 40 + HI groups (D). Scale bar: 100 µm. Data are expressed as the mean ± standard deviation (*SD*). *p* value: *p < 0.05, **p < 0.01, Where ‘a’ represent the comparison with the sham control group; ‘b’ represent the comparison with the HI-insulted group.

**Figure 6.**
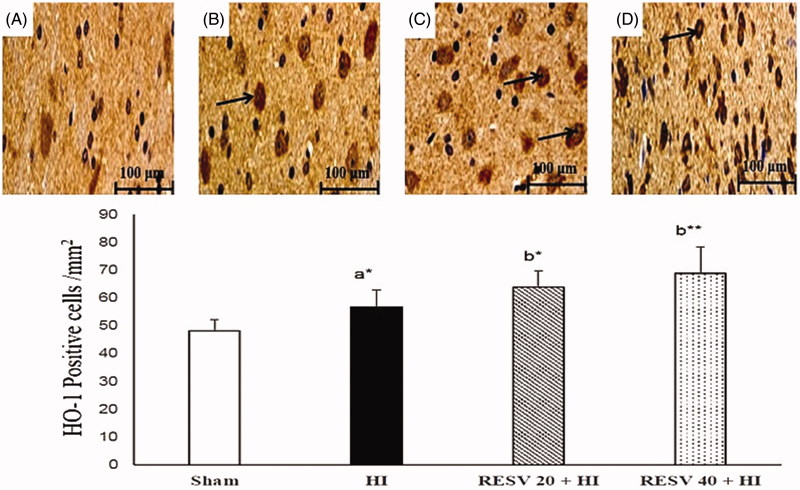
Efficacy of RESV on the immune-reactivity of HO-1 (A–D) in the cerebral region of experimental neonatal rats. In the sham group, only a few cells were positively stained for cytosolic HO-1 (A). In cerebral tissues (infarct regions) of HI-induced rat brains, showed increased positively stained cells for cytosolic HO-1 (B). In the RESV 20 + HI (C) and 40 + HI groups (D), a furthermore increase in the staining for cytosolic HO-1 was observed (indicated by arrows). Scale bar: 100 µm. Data are expressed as the mean ± standard deviation (*SD*). *p* value: *p < 0.05, **p < 0.01, Where ‘a’ represent the comparison with the sham control group; ‘b’ represent the comparison with the HI-insulted group.

Few positively stained cells of Nrf2 and HO-1 are detected in the sham group. Increased immunoreactivity (*p* < 0.05) of nuclear Nrf2 (Figure 5) and cytosolic HO-1 (Figure 6) were noted in cerebral (infarct regions) of the HI-induced brain than sham control rats. However, pretreatment with RESV (20; *p* < 0.05 and 40; *p* < 0.01) exhibit a further increase in the intensity of nuclear Nrf2 and cytosolic HO-1 positively stained cells, indicating increased translocation of Nrf2 was favored by RESV. The result of immunohistochemistry (IHC) was concordant with the outcome of western blot. RESV 40 treatment group showed better neuroprotective efficacy in lowering oxidative stress and inflammatory response than RESV 20 group by upregulating the protein expression of cytosolic HO-1 and nuclear Nrf2.

## Discussion

The current study is the first animal experiment conducted to explore the neuroprotective effects of RESV against HI-induced brain injury by evaluating Nrf2/HO-1 signalling pathway. RESV is a naturally occurring non-flavonoid polyphenolic compound which has been highlighted for its several biological properties including antioxidant, anti-inflammation and anticancer, as well as cardio- and neuroprotective effects (Guo et al. 2015; Feng et al. 2016). Reports show that oxidative stress and subsequent inflammatory reactions after HI insult are the major contributors for the pathophysiology of HIE (Lu et al. 2012; Garcia-Bonilla et al. 2014; Arteaga et al. 2014). The neonatal brain is more vulnerable to oxidative stress (damage) more than the adult brain owing to lower levels of antioxidants, higher oxygen utilization rate and a high content of PUFA, which are more susceptible to oxidant (free radical) generation, which may cause neural damage (Fukui et al. 2002; Buonocore and Groenendaal 2007).

Brain infarction models may be utilized for assessing the degree of neuronal damage under cerebral ischemic conditions (Yousuf et al. 2009). In the present study, the HI-induced group developed a large infarct area and oedema (water accumulation) as well as neuronal damage and inflammatory response. Himadri et al. (2010), demonstrated that increased oxidative stress during HI insult could considerably downregulate Na^+^-K^+^-ATPase, which in turn trigger oedema via altering BBB. Pre-treatment with 20 and 40 mg RESV lowered the infarct area and oedema by suppressing oxidative stress and inflammatory cytokines owing to its antioxidant and anti-inflammatory potential. Our results are in agreement with the results of Ren et al. (2011), who inferred that RESV effectively reduces the infarct volume and oedema after focal cerebral ischemia. Moreover, RESV treatment significantly decreased the infarct area by the improved preservation of myelination (Karalis et al. 2011).

The viability and morphological changes of neurons were evaluated by Nissl staining, where cresyl violet (primary stain) was used to visualize the cytoplasm of neurons, including Nissl corpuscles. Nissl bodies (staining with cresyl violet) are an indicator of neuronal integrity and may be lost during neuronal damage, which aids in determining the number of viable neurons (Telles et al. 2014). Marked neuronal changes were observed in HI insulted rats with increased pyknotic neurons and less visible Nissl bodies (increased neuronal death). Compared with the HI group, the RESV treatment groups displayed a lesser extent of alterations in cell morphology and an increased number of Nissl bodies (viable neurons), indicating the neuroprotective ability of RESV by actively scavenging free radicals.

The antioxidant defense system in our body is composed of enzymic and non-enzymic antioxidants to scavenge excessive free radicals. SOD, CAT, and GPx are the main enzymic antioxidants which maintain the balance between pro- and antioxidants (Muralidharan et al. 2008). SOD enhances the conversion of superoxide (O_2_^−^) to hydrogen peroxide (H_2_O_2_), whereas GPx and CAT catalyze the reduction of H_2_O_2_ to water (Kamesh and Sumathi 2014). HI-induction in neonatal rats triggers the excessive ROS generation (activation of NADPH oxidase or xanthine oxidase) owing to increased oxidative stress. Thus, to eliminate these excessive free radicals, the immune system of HI-induced rats triggered the Nrf2-dependent pathway to upregulate the synthesis of endogenous antioxidants enzymes such as GPx, SOD and CAT to quench the free radicals and hence, the antioxidant activities in the HI group were considerably improved than sham group. In addition, the levels of the lipid peroxidation product MDA were markedly increased in the HI group due to oxidative stress.

Treatment with RESV (20 and 40 mg/kg) substantially increased the activities of GPx, SOD and CAT as well as dramatically attenuated HI-induced increases in the MDA levels in the cerebral cortex homogenate. RESV ameliorate the endogenous antioxidant activities in HI-induced rats to abolish the oxidative stress by activating the Nrf2/HO-1 pathway (Das et al. 2006; Pallas et al. 2013), but the exact mechanism remains elusive. Moreover, RESV is demonstrated to inhibit xanthine oxidase enzyme and thus halt the production of hypoxanthine and free radicals under ischemic condition (Li et al. 2011). RESV can scavenge a variety of free radicals owing to its phenol rings and three free hydroxyl group, and thus RESV treatment can concomitantly abrogate the free radical production under ischemic conditions (Sinha et al. 2002; Simao et al. 2011).

Inflammation is the main contributor to HI brain damage. In line with this, the present study showed that the levels of pro-inflammatory cytokines, including IL-6, IL-1β, TNF-α and NF-κB free p65 were markedly higher in rats following HI induction attributing to overproduction of free radicals, which results in enhanced production of numerous cytokines (inflammatory cascade). These pro-inflammatory cytokines induce microglial activation and thereby favour the production of further cytokines and a subsequent inflammatory cascade (Cowell et al. 2002; Block et al. 2007). Compared with rats in the HI group, pretreatment with RESV (20 and 40 mg/kg) significantly suppressed the levels of these pro-inflammatory cytokines, thus suggesting its anti-inflammatory activity. These results were supported by Shin et al. (2012), demonstrated that acute RESV administration during ischemic conditions successfully suppressed brain injury in mice by lowering the expression of (IL-6, IL-1β and TNF-α in the ischemic cortex. RESV pretreatment markedly diminished the activation of microglia and astroglia (astrocytes) during cerebral ischemia, which was accompanied by an inhibition of NF-κB activation (Renaud and Martinoli 2014). Wang et al. (2002), demonstrated that RESV could enhance the cerebral flow in gerbils by crossing BBB to exert a protective effect against cerebral ischemic injury by inhibiting glial cell activation and thus halt the inflammatory response. Therefore, RESV treatment had a significant impact on the antioxidants status and various inflammatory markers, indicating its antioxidative and anti-inflammatory efficacy against HIE.

The precise mechanisms of how resveratrol alleviates HI-induced oxidative stress and inflammatory responses in rat pups remain elusive. However, it may be speculated that RESV triggers the Nrf2/HO-1 signalling pathway. Previous studies have reported that activation of Nrf2 may have a crucial role in triggering the endogenous defense system, thus protecting neural cells to inhibit ischemic brain injury (Wang et al. 2002; Guo et al. 2014). The protein expression of nuclear Nrf2 and cytosolic HO-1 were assessed by western blot analysis and IHC to confirm the neuroprotective capacity of RESV. The results of the two assays showed a similar trend, namely, a slight/mild increase in Nrf2 (nuclear) and HO-1 (cytoplasmic) in the HI-induced group, presumably due to increased oxidative stress evoking an immune response to trigger the Nrf2/HO-1-dependent pathway to produce the endogenous antioxidants, namely, GPX, SOD and CAT to quench the free radicals. Furthermore, HO-1 may indirectly act as an antioxidant by catabolizing heme to biliverdin (exerting antioxidant effects), iron and CO (using anti-inflammatory effects) as well as stimulate Nrf2 activation (Tanaka et al. 2007; Zhang et al. 2014).

**Figure 7.**
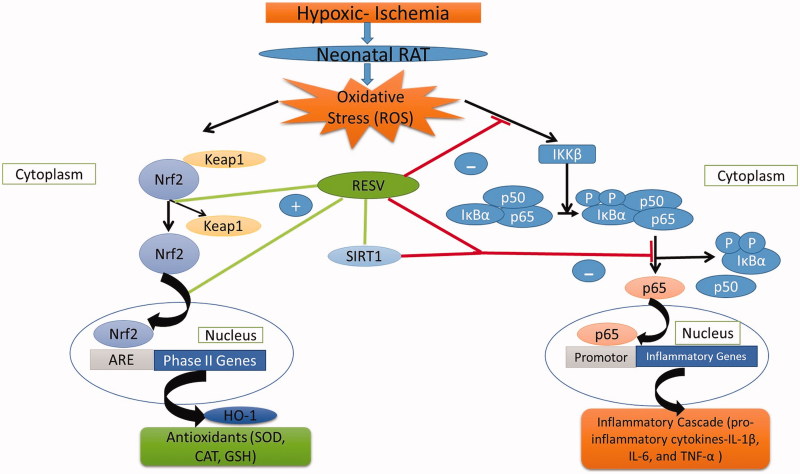
The schematic representation of mechanisms of action of RESV against HIE.

RESV-treated rats showed a further increase the expression of cytosolic HO-1 and nuclear Nrf2 on equivalence to HI-induced rats. Numerous studies have reported that RESV upregulates ARE expression via cleaving the Kelch-like ECH associated protein 1 (Keap 1)-Nrf2 complex by activating Akt/PI3K and MAPK (p38) signalling pathway to release and activate Nrf2 (Shin et al. 2012; Kodali et al. 2015). The activated Nrf2 translocates from the cytoplasm to the nucleus and binds to the ARE promoter region to positively upregulate the expression of cytoprotective factors like HO-1, which might be the reason for the upregulation of Nrf2 and HO-1 protein expression in RESV-treated rats. Thus, the results of the present study demonstrated that RESV acts as an activator of Nrf2. Another study also hinted that RESV could indirectly regulate SIRT1 expression and thus blocks the translocation of activated NF-p65 subunit from cytosol to the nucleus and thereby halting the production of various inflammatory markers (cytokines) including IL-6, IL-1β, and TNF-α (Csiszar 2011). Furthermore, owing to the increased endogenous antioxidant activation by RESV, it potentially scavenges free radicals (ROS) and thereby inhibits the activation of the inflammatory cascade. The precise mechanisms by which RESV alleviates oxidative stress and its subsequent inflammatory response are depicted in Figure 7.

Of note, the present study had certain limitations. For instance, the focus was on only the acute neuroprotective effects of RESV treatment in the brains of rats subjected to HI insult, while the long-term (chronic) impact of RESV are yet to prove as well as the interlink between various signalling pathways associated with Nrf2/HO-1 was not evaluated. Furthermore, the mRNA expression of endogenous antioxidants related to Nrf2 and HO-1 as well as inflammatory markers were not assessed, which would have strengthened the results regarding the levels of their proteins (the end products of mRNA). In future studies, the effects of pre- and post-HI treatment with RESV will be assessed. While the present study focused on oxidative stress and inflammatory response and hence we did not determine apoptosis, glutamate excitotoxicity and mitochondrial dysfunction as well as its association with bioenergetics, even though they are major contributors to HIE, which would be addressed in future studies.

## Conclusions

The present study demonstrates that the protein expression of HO-1 and Nrf2 was positively modulated by RESV treatment in neonatal rats subjected to HI, which may have contributed to its neuroprotective activity, with corresponding decreases in infarct volume and cerebral oedema, elevated levels of antioxidants, suppression of inflammatory markers and improved neuronal morphology. RESV may be utilized as a neurotherapeutic agent, which acts through upregulation of the Nrf2/HO-1 pathway and has a multifaceted potential to attenuate hypoxia-induced ischemic brain damage in neonatal rats by inhibiting oxidative stress along with the inflammatory response. The possible involvement of other pathways (Akt/PI3K and MAPK) warrants further investigation to reveal the detailed mechanisms of the neuroprotective effects of RESV.
